# Numerical parametric optimization of fluid flow profiles in membranes using the Taguchi method

**DOI:** 10.1038/s41598-025-19792-z

**Published:** 2025-10-14

**Authors:** Muhammad Arslan, Haizhen Xian, Imran Shah, Shahid Aziz, Dong-Won Jung

**Affiliations:** 1 School of Energy Power and Mechanical Engineering, North China Electric Power University, Beijing, 102206 China; 2 Department of Mechatronics Engineering, Air University, Islamabad, 44000 Pakistan; 3 Department of Mechanical Engineering, Jeju National University, 102 Jejudaehak-ro, Jeju-si, 63243 Republic of Korea; 4 Faculty of Applied Energy System, Major of Mechanical Engineering, Jeju National University, 102 Jejudaehak-ro, Jeju-Si, 63243 Republic of Korea

**Keywords:** Taguchi method, Optimization, Membrane modeling, COMSOL 6.3, And fluid flow, Engineering, Mathematics and computing

## Abstract

Membrane modeling and flow through it are important studies, and their applications are for gas and water separation. The examples of membrane modeling applications are artificial kidneys and reactors. Some studies are available on membrane modeling for different applications; however, optimization studies are rarely available. In the current study, an initial membrane model is simulated by COMSOL 6.3 and then optimized for input parameters to decide the optimal set at which the performance and flow profiles are better. For this study, the design of experiments table through Minitab software using the Taguchi method is generated. Then it tested for an optimal parametric selection. In this study, the focus is on the effect of dialysate concentration (D_c_), partition coefficient (K), dialysate velocity (D_v_), and permeate velocity (P_v_) on concentration individually and then in combination. The individual effect of parameters with different values is first studied. Then an array of L_16_ (4×4) using the design of experiments method (DOE) concept is finalized for variance analysis (ANOVA) to study the combined effect. The result of this analysis concludes that the major impact on concentration and velocity profile is due to K. The combined effect results conclude that the effect of different parameters is in the order of K > P_v_ > C_d_ > D_v_ for concentration and velocity profiles. This means that the major effect is due to the partition coefficient, and the minor effect is due to the permeating velocity. Before testing new results, the current COMSOL model is validated against published experimental work to ensure its accuracy.

**Supplementary Information:**

The online version contains supplementary material available at 10.1038/s41598-025-19792-z.

## Introduction

The membrane study is very important for gas separation^1,2^, hemodialysis^3^, wood drying process^4^ inorganic purification, absorption^5,6^, freshwater production^7^, artificial kidneys^8^, fuel cells^9^ filtration^10^, hydrogen production and purification^11^, chemical removal like ammonia^1^ and protein exchange membrane fuel cells^12^. The membrane may be hydrophobic, porous^13^, or hydrophilic. The type is selected as per the requirements, conditions, and application^14^. Numerical membrane modeling provides predictions for solvent absorption, adsorption, and cryogenic fractionation. These are the traditional methods used for the purification and separation of different gas mixtures and agree with experimental work in real-time scenarios^15^. The membrane distillation system can be classified in four different ways. These four are called a conventional direct-contact membrane distillation system^16^air gap membrane distillation^17^, vacuum membrane distillation^18^, and sweeping gas membrane distillation^19^. Membrane distillation is believed to be an emerging technology for desalination and wastewater treatment purposes^20^. The membrane distillation research opened new windows for research in the flat sheet and hollow membranes. In such kinds of membranes, thermal energy efficiency is enhanced by minimizing thermal polarization, improving heat recovery from condensed vapor, and increasing mass transfer across the membrane^21^.

Dai et al.^22^ studied CO_2_ capture by using an ionic liquid passing through a tubular membrane structure. They concluded from their study that the membranes having high porosity and small thickness offer low membrane resistance, which results in separation efficiency improvement. Taghvaie et al.^23^ worked on CFD modeling of CO_2_ separation using a tubular membrane with absorbents of EDA, PS, and PZEA. They concluded that the inlet CO_2_ sequester of 88.75% is that of PZEA. Baghel et al.^24^ presented CFD COMSOL 6.3 modeling for vacuum membrane distillation and tried the application of naphthol blue-black dye removal from the aqueous solution. They concluded that the temperature polarization coefficient (TPC) reduced from 0.81 to 0.48, resulting in an increase of feeding temperature to a 25–85 °C range. This effect reduces the heat transfer process, which further leads to a reduction of permeate flux. Maqbool et al.^25^ worked on the modeling and simulation of direct contact membranes using sunlight thermal energy and freshwater. They concluded that the evaporation efficiency of PV/T-DCMD increases as temperature rises to 42.01% from 35.08%. A similar study was done by Samadi et al.^26^and concluded some optimal values for the dimension of a 1m^2^ membrane. Also, some optimal set of input parameters like temperatures more than 20 ^0^C, concentration less than 1000 mol/m^3^, and feed velocity in the range of 1–1.15 mm/s is selected. The overall gain is 70% as per the results and analysis observed at these optimal conditions. Kadi et al.^27^ worked on direct membranes with and without spacer designs, first investigated numerically and then compared with experimental work. They concluded that better results are possible in the case of the D1.5 and D3.5 models for a simple model without spacers.

Membrane modeling is an important research area in which different researchers have tried numerical models for the fluid flow through a membrane. Some researchers even tried their experiments for different applications along with numerical modeling. However, very few studies are available on the optimization of fluid flow through membranes at the micro- and nanoscale for better design prediction. As per the author’s knowledge, there are a few research articles available on the optimization of fluid flow for input parameters by design of experiments using the Taguchi method. That is why in this study, the focus and novelty are to optimize the design for input parameters for future modeling and experimentation. This study starts with the initial basic model and results. Then, the different input parametric values are tested through COMSOL 6.3 software to see the effect each parameter has on the concentration profile individually. Lastly, for checking the combined effect of all parameters at the same time, the design of the experiments table is generated for an array of L_16_ (4×4) using Minitab software for ANOVA analysis using the Taguchi concept. The analysis based on the DOE table results in a conclusion of the order of rank-wise. This study will help in selecting the right parameters for real-time applications and studying through membranes.

## Design and methodology

**Fig. 1:**
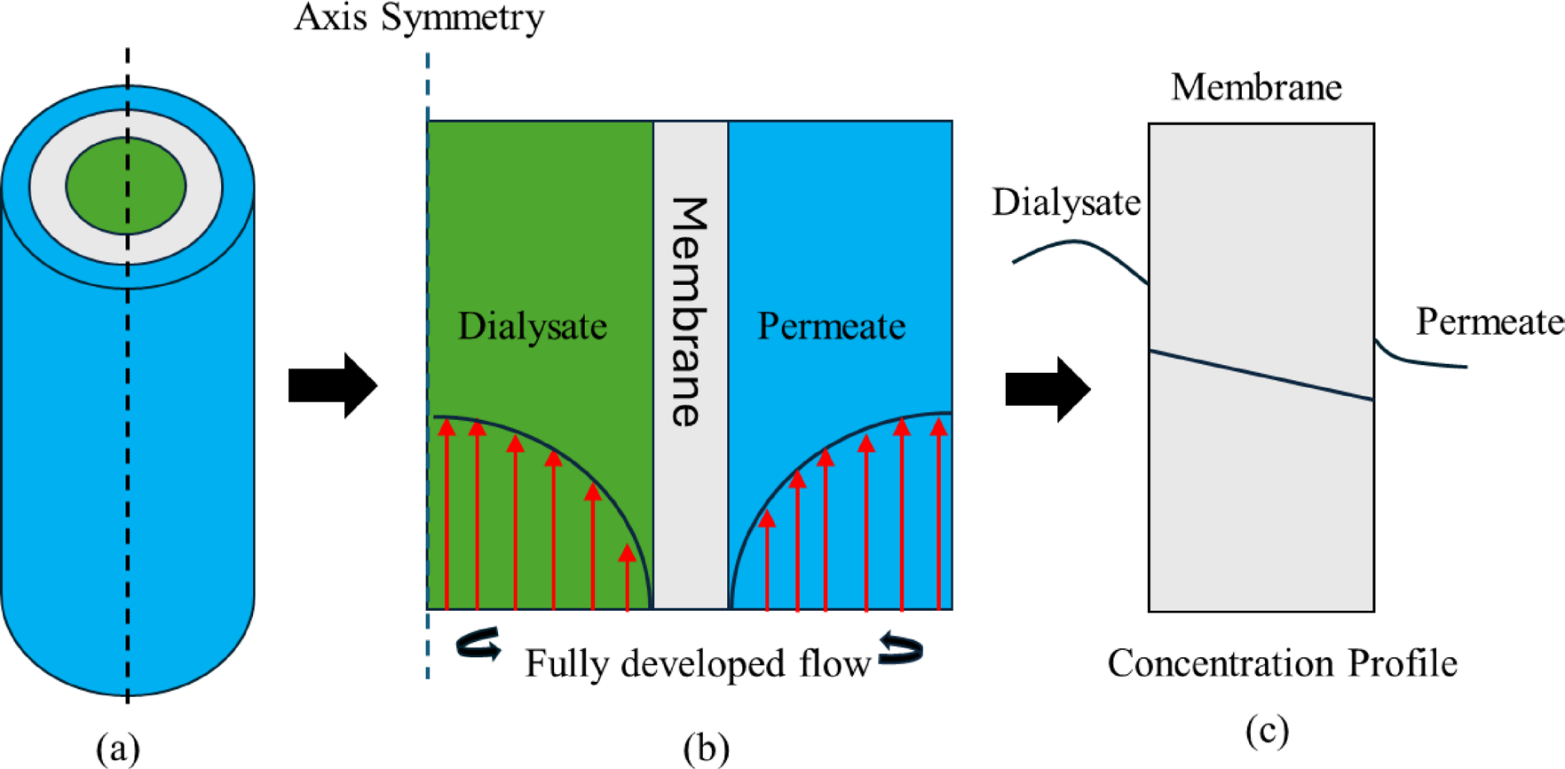
Schematic diagram of membrane modeling (**a**) 3D cylindrical view of membrane fiber. (**b**) Fully developed flow through the 2D model using the axis symmetry concept. (**c**) 2D Concentration profile after the fluid passed through the membrane.

Figure 1 represents the design and methodology steps for direct membrane modeling simulation. In this work, the 3D model is simulated using the axis symmetry concept in 2D form to save computational time and cost. The results are presented and interpreted in 2D and 3D views. Through the revolve command, 2D results can be easily converted to 3D in post-processing.

**Fig. 2:**
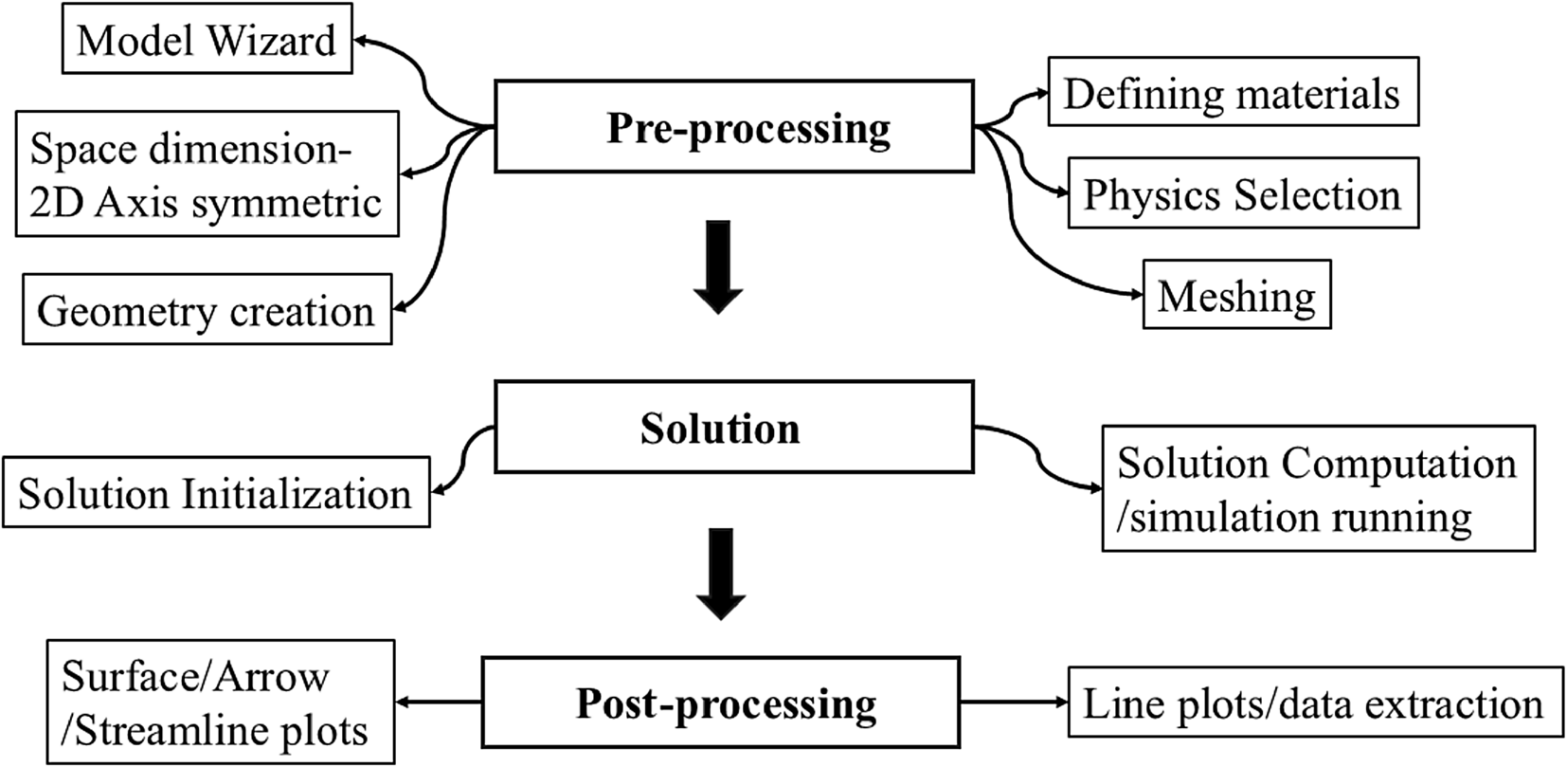
The Flow chart for the simulation model consists of three steps: pre-processing, solution, and post-processing.

The general flowchart used for the simulation process consists of three parts: preprocessing, solution, and postprocessing. Preprocessing usually consists of geometry, physics, boundary conditions, material properties, and meshing creation. In the solution part, the solution is initialized with initial values, and then the computation is run to find the solution of the computational domain. During computation, the finite element method-based schemes programming run codes in the background of the software as per the assigned physics and boundary conditions. Once the solution is obtained, then postprocessing is done by extracting data from COMSOL 6.3. This data is in the form of figures, surface, arrow, and line plots. Figure 2 is the summary of these points in the form of a flow chart. Table 1 represents the geometric and initial input parameters.

**Table 1:** Represents the geometric and initial input parameters.

Name	Expression	Unit	Description
R_i_	0.2	mm	Inner radius
L_t_	0.08	mm	Membrane thickness
L_pc_	0.42	mm	Width of the concentric permeate channel
H	21	mm	Fiber length
D	1.00E-09	m^2^/s	Diffusion constant for liquid phases and membrane
K	0.7		Partition coefficient
C_0_	1	mol/m^3^	Dialysate inlet concentration
C_1_	0.1	mol/m^3^	Permeate inlet concentration
C_2_	0	mol/m^3^	Membrane initial concentration
U_ave_di_	0.5	m/s	Dialysate average velocity
U_ave_pe_	0.8	m/s	Permeate average velocity

## Validation of simulation model

**Fig. 3:**
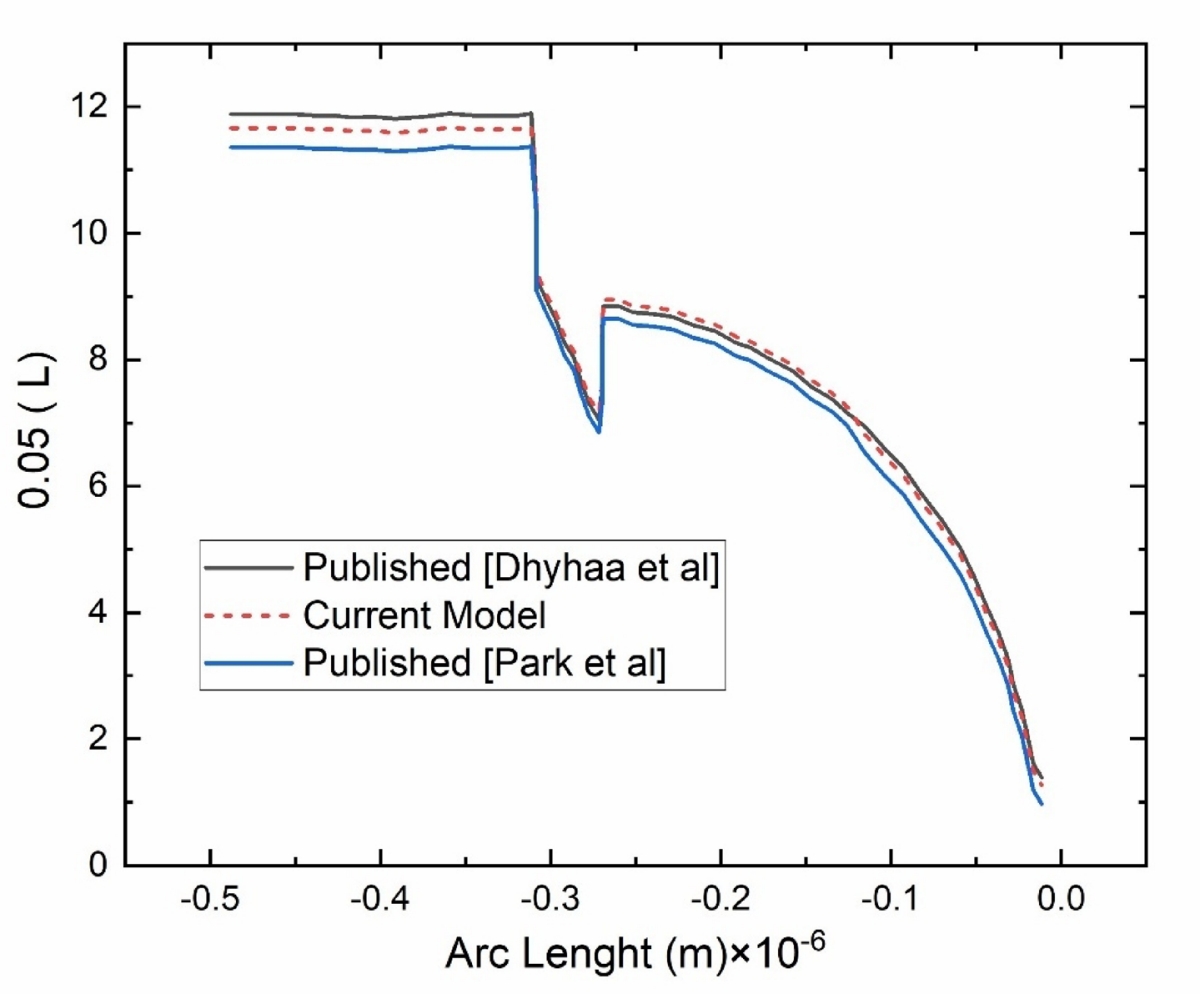
Validation of results with recently published articles of Dhyhaa et al.^15^ and Park et al.^28^.

For the simulation model, it is compulsory to validate it against published results. Therefore, the current model is validated against the published results of Dheyaa et al.^15^ and Park^28^ et al. as shown in Fig. 3. The same geometry is first designed in COMSOL 6.3 according to the dimensions mentioned, then the physics and the same boundary conditions are applied to replicate the results for the current model. The researchers Dheyaa et al.^15^ presented membrane-related work both numerically and experimentally, while the Park et al.^28^only numerically with experimental validation evidence. The results of the current model obtained are in good agreement with the published work. Hence, this validated model can now be implemented to get new results for the proposed work. The qualitative results comparison is shown in Fig. 3.

The quantitative results dashed lines are close with the published articles’ results. The same validated COMSOL 6.3 model is used for the current work with new parameter values and a design of experiments table as an input to do the optimization of input parametric fluid flow through the membrane. The % error analysis of the current proposed model and published papers is summarized below in Table 2. The results show good agreement as the % error is below 5% for all values.

**Table 2:** shows the % error analysis of the current model and published papers.

	Dheyaa et al.^15^	Park et al.^28^	Current model	% Error of current model with^15^	% Error of current model with^28^
Reading#01	11.85	11.33	11.63	1.89	2.57
Reading#02	7.86	7.66	7.96	1.25	3.76
Reading#03	3.25	2.83	3.13	3.83	4.79

## Numerical modeling and simulation

### Physics and boundary conditions

To solve this problem, COMSOL 6.3 uses two modules: laminar flow and diluted concentration. These two modules are coupled together for the solution. The Dirichlet boundary conditions are used at the inlets and outlets of the computational domain. The inlet velocity for the initial model is 0.1 m/s, and then it varies for the optimization model. In the same way, zero pressure at outlet conditions is applied. The fluid flow study is governed by the physics of three-dimensional steady and incompressible systems of Navier–Stokes^29^ coupled together with Fick’s law of diffusion through the membrane for quantification of concentration variations. Equations (1–4) are represented below.

1 $$\uprho \left(\text{u}\cdot \nabla \right)\text{u}=\nabla \cdot \left[-\text{pI}+\text{K}\right]+\text{F}$$

2 $$\uprho \nabla \cdot \text{ u}=0$$

3 $$\nabla \cdot {\text{J}}_{\text{i}}+\text{u}\cdot \nabla {\text{C}}_{\text{i}}={\text{r}}_{\text{i}}$$

4 $${\text{J}}_{\text{i}}=-{\text{D}}_{\text{i}}\nabla {\text{C}}_{\text{i}}$$

where D_i_ is the diffusion coefficient, u = velocity, F = body forces, C_i_ = Concentration, r_i_ = reaction source, and p = pressure. The fluid after passing through the membrane with a certain velocity has a high concentration, as indicated by the red color. It decreases as the fluid passes through a membrane with an increase in speed due to the diffusion and convection processes. There is a reduction in concentration changes indicated by blue and green in the color legend. These four physics equations are used to solve the problem. However, these are in generic forms used on the back of the software. In setting the model inside COMSOL 6.3, certain assumptions are used, i.e., fluid flow is incompressible, convection is an option selected during laminar flow physics, etc. The fluid flow is laminar because of the small dimensions calculated and indicated through the Reynolds number. The membrane is assumed to be uniform and flawless using the continuum assumption of mechanics. The model is predictive for results without the wettability and the rough surface effect of the membrane. The study is carried out in steady state conditions, as after a certain time, there are no major changes in results with the passage of time.

### Mesh independence study

**Fig. 4:**
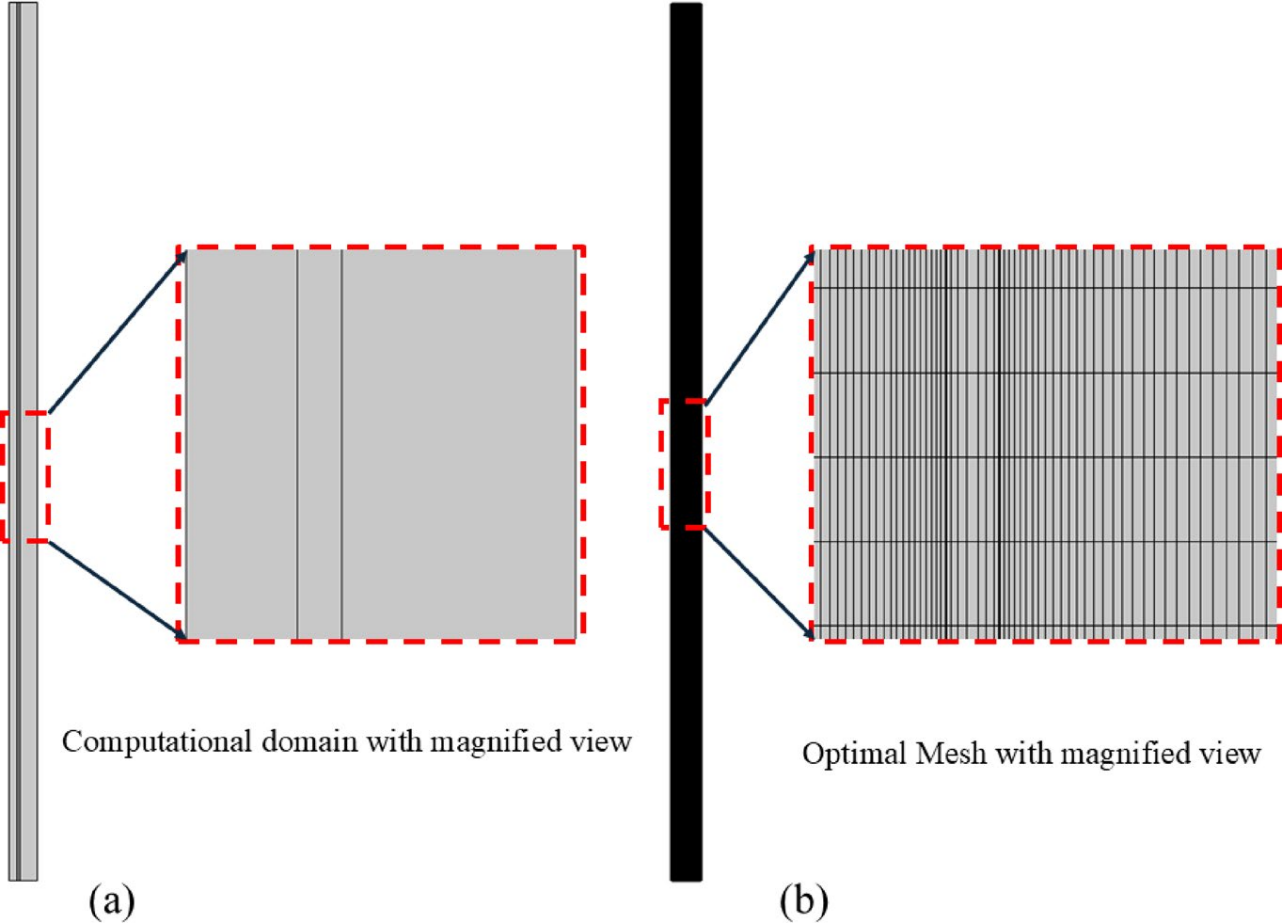
(**a**) Shows the computational domain with a magnified view, and (**b**) the optimal mesh along with mesh statistics with a magnified view for better visualization.

For the simulation model, finding the optimal mesh by the mesh independence method is a compulsory requirement. The optimal mesh can be defined as “the mesh with the least number of elements for computation but with enough accuracy.”^30^. In this method, the computation starts with normal discretization, and then different cases with refined mesh are computed until the mesh change has no more major effects on the results^31^. The model is said to be mesh or grid-independent^32^ and the accuracy of results is ensured. Figure 4 represents the mesh independence test results for this study, along with the optimal meshing statistics.

**Fig. 5:**
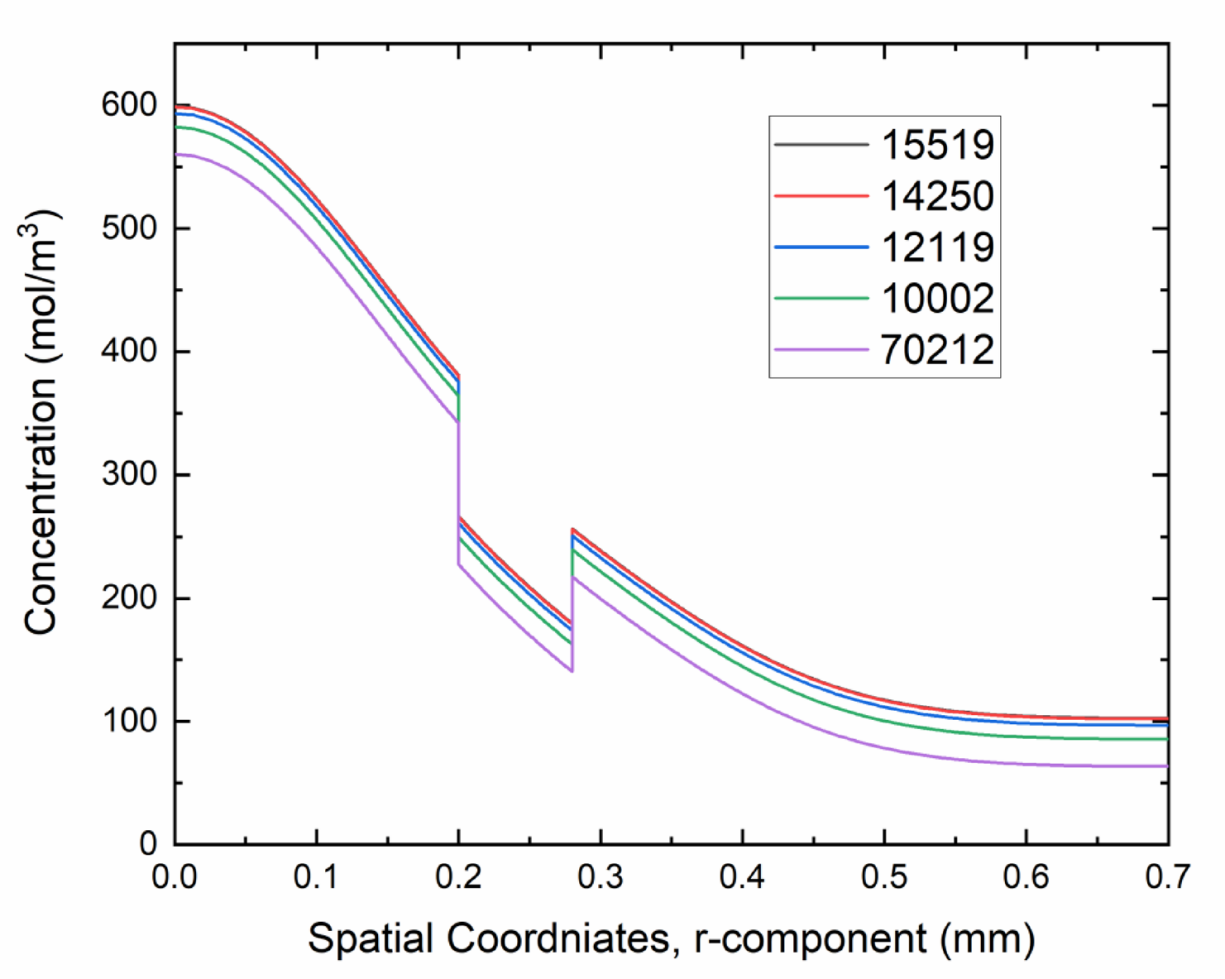
Shows the mesh independence test for the optimal mesh selection. The mesh with the number of elements 14,250 is the optimal mesh for simulation results.

Figure 5 shows the mesh independence line plot. This plot is compulsory for the accuracy of results, so that with a change in mesh, there is no major change in results. For this, the computation started with a mesh consisting of 70,212 number of elements. The number of elements can be easily checked by reading the mesh statistics in COMSOL 6.3. From mesh statistics, besides the number of elements, there are details of the vortex, edge elements, etc. Further refining of the mesh until the results became stable ended with 15,519. The optimal mesh was decided based on this testing on 14,250 number of elements. The edge elements in optimal are 1114, and the vertex elements are 8. This is because the results for the number of elements 14,250 and 15,519 are too close to show any major change in results. The mesh with 14,250 is selected as an optimal mesh to save computational time, as the accuracy of the results is enough.

### Velocity and concentration surface plots

**Fig. 6:**
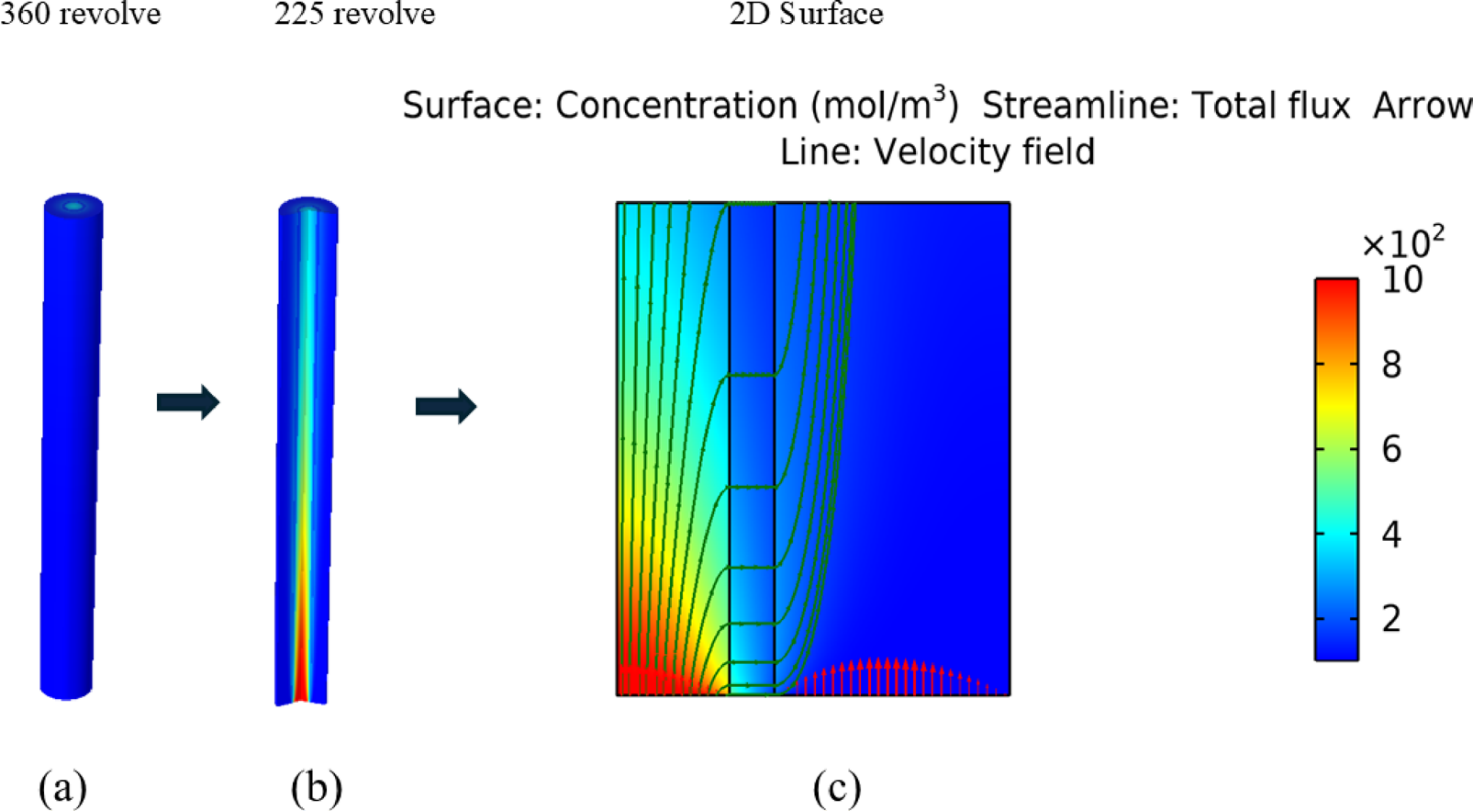
(**a**) 3D revolution view, (**b**) 225 views for showing internal structure profile, (**c**) 2D Surface velocity and concentration surface plots, arrow lines.

**Fig. 7:**
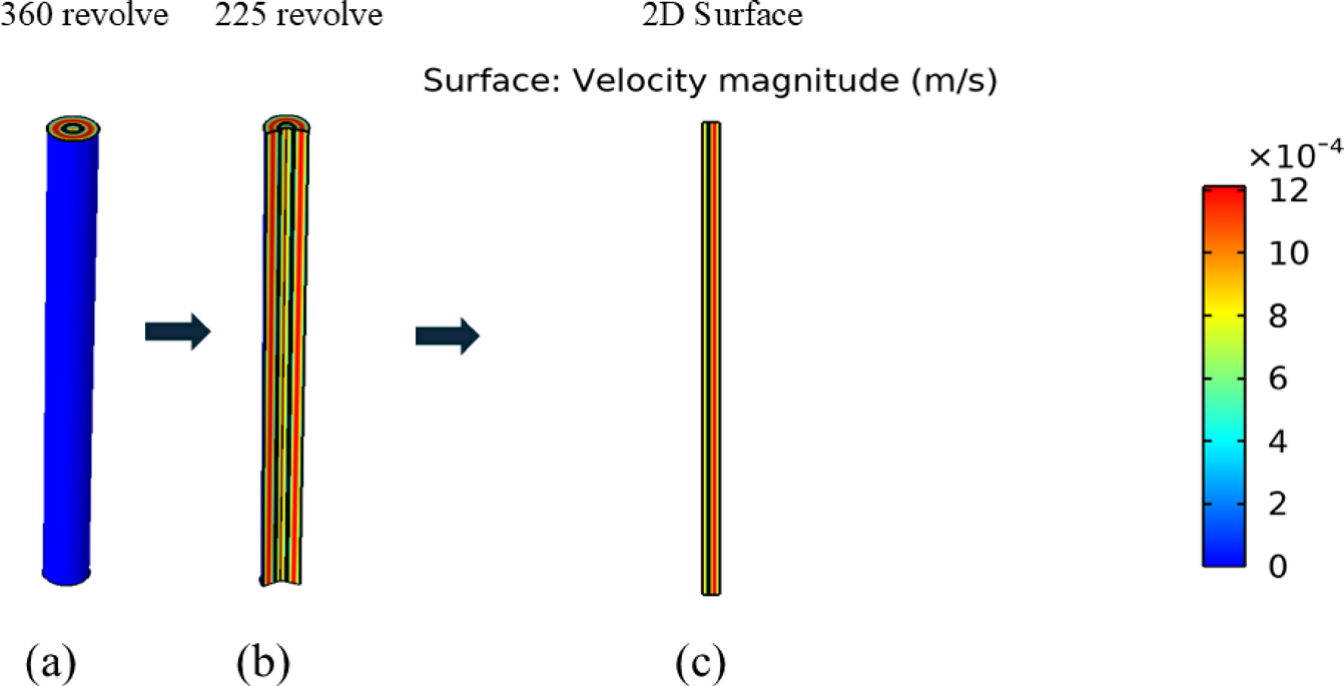
(**a**) 3D revolution view, (**b**) 225 views for internal geometry profile, (**c**) 2D Surface velocity.

The concentration and velocity surface plots for fluid flow through the computational domain are respectively represented by Figs. 6 and 7. The concentration is high at the middle and low at the outer edges of the circular 3d membrane. For clearer 2D surface plot visualization, an arrow is presented, which confirms that concentration is higher at the center of the cylindrical membrane layer.

The velocity varies across the different layers of the cylindrical membrane portions. In the center, it is at a medium level and after that increases to the highest level as indicated by red color, and finally decreases again.

### Velocity and concentration plots

**Fig. 8:**
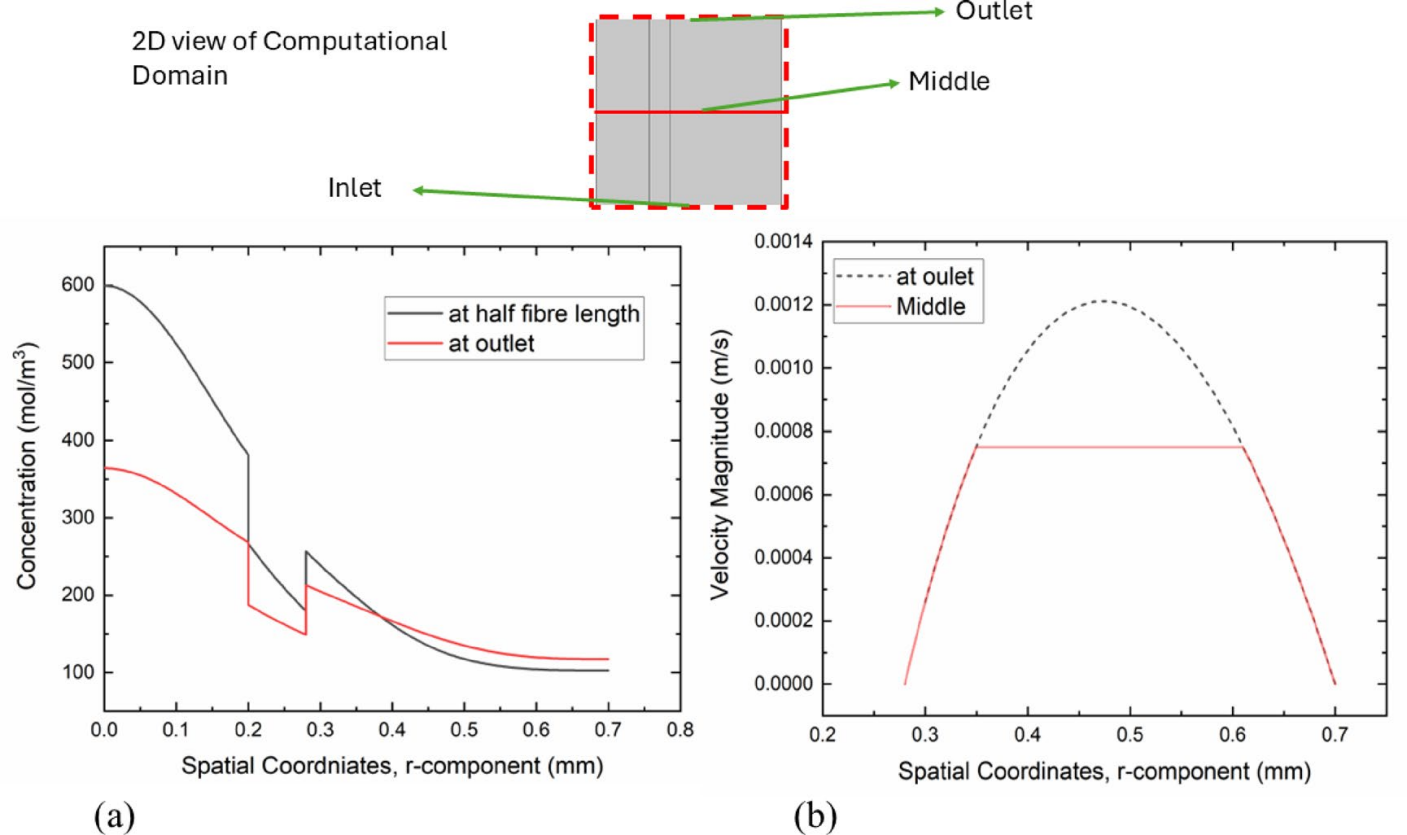
(**a**) Shows the concentration profile at the inlet and outlet, and (**b**) shows the velocity profile through cut lines at the middle and outlet of the computational domain.

The figure represents the results for velocity and concentration profile through a cut line passing through the inlet, half fibre length, and the middle of the computational domain. Figure 8a shows that the concentration profile changed as the flow passed through the inlet, streaming up towards the top. The concentration at half fiber length and outlet is presented. Figure 8b shows fluid speeds when passed from the inlet to the outlet, and the speeds observed until fully developed flow. It is noted that at the inlet, there is more resistance due to no-slip conditions near the walls. The fluid profile is not fully developed in the middle. As fluid reached the outlet, it became a fully developed flow profile.

## Optimization

### Effect of dialysate concentration

**Fig. 9:**
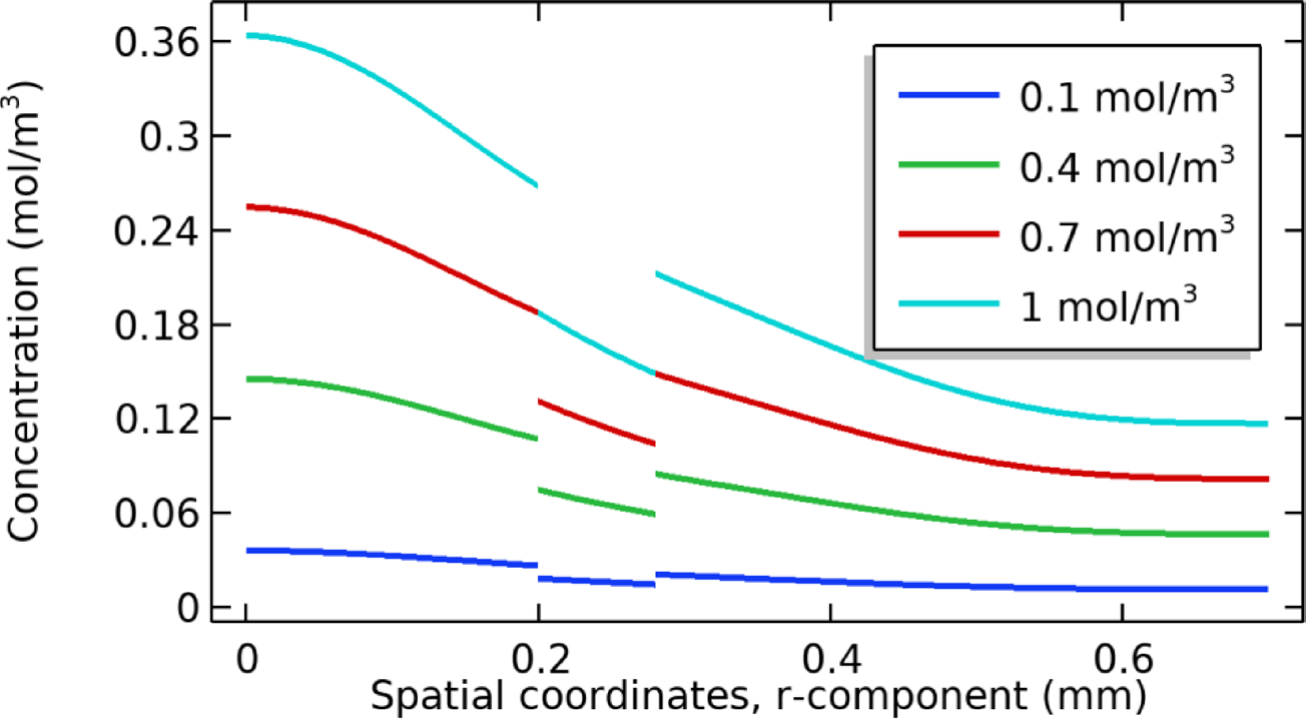
Shows the effect of the concentration of dialysate on the overall concentration profile.

The dialysate concentration values are changed from 0.1 to 1 mol/m^3^ to check their effect on the concentration profile as fluid passes through the computational domain. The overall concentration of fluids is trending high at high values and vice versa. The results are presented in Fig. 9, shown below.

### Studying the effect of partition coefficient

**Fig. 10:**
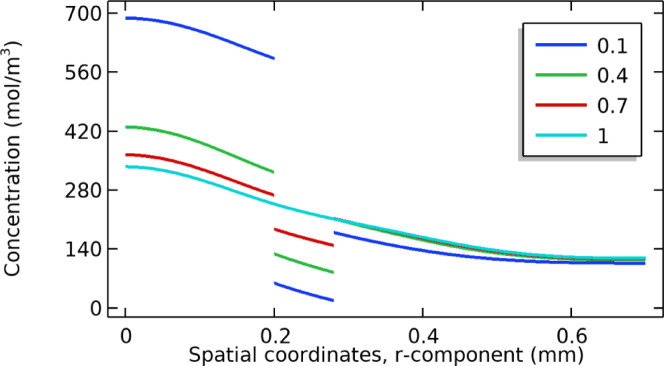
Shows the effect of the partition coefficient on the concentration profile.

The partition coefficient is a vital ratio of concentration for input at different regions of the computational domain, especially at the junction region. To study the effect of this parameter, the values are changed from 0.1 to 1. The results of this parameter analysis are shown in Fig. 10. This line plot reveals that by increasing the value of k, the concentration values decrease in all parts of the computational domain. However, the effect due to K at different regions varies in magnitude. The reason for its physical interpretation is that the partition coefficient is stronger near the feed and membrane interface. And it is different in other parts of the computational domain due to variable mass transfer. After a certain time, the concentration drops due to the uptake phenomenon inside the membrane, leading to a nearly uniform concentration profile. These results agree with the theory explained in the experimental research done by Farah et al.^33^ very early.

### Effect of dialysate velocity

**Fig. 11:**
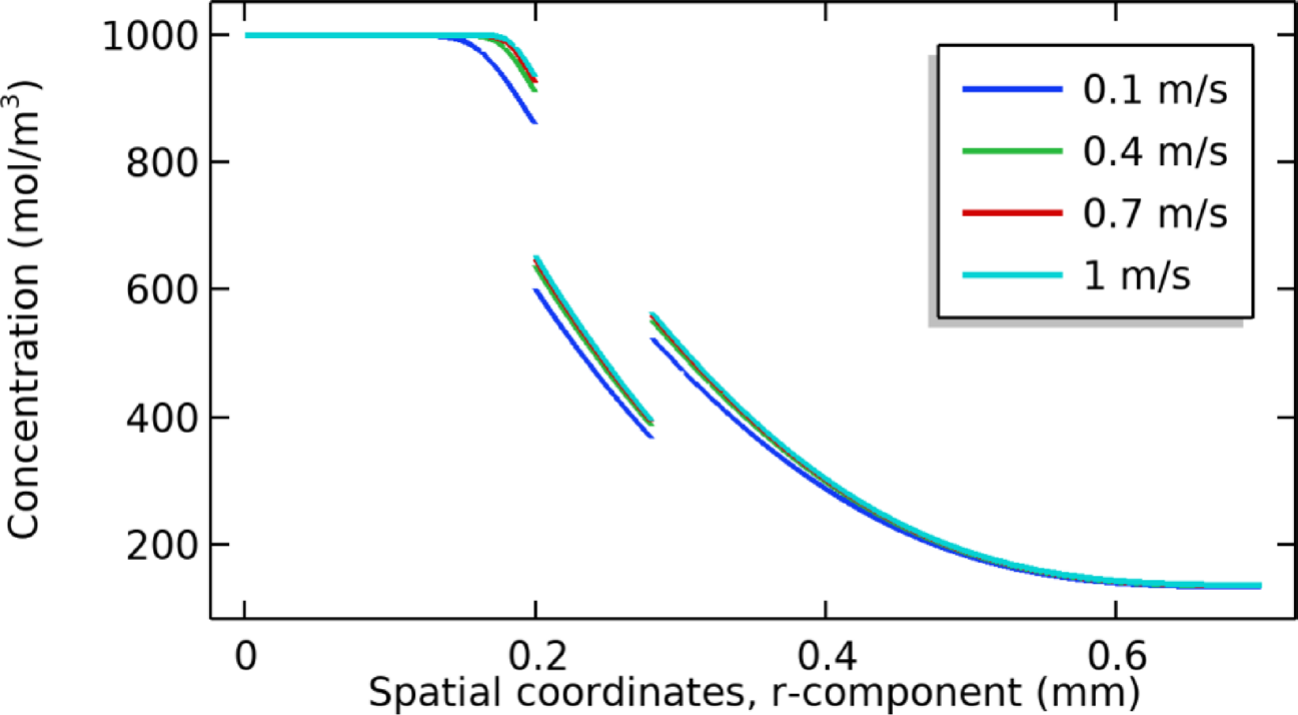
Shows the effect of dialysate velocity on the concentration profile.

The effect of the dialysate velocity is studied by changing its values from 0.1 to 0.4 m/s. The results show that by increasing the velocity, fluid passes quickly through the computational domain, resulting in a decrease in concentrations of dialysate and membrane portions. These results are presented in Fig. 11. It is observed that speed has no major effect on the permeate part and its concentration profile remains the same or closes with minor changes. The overall trend is decreasing in nature.

### Effect of permeate velocity

**Fig. 12:**
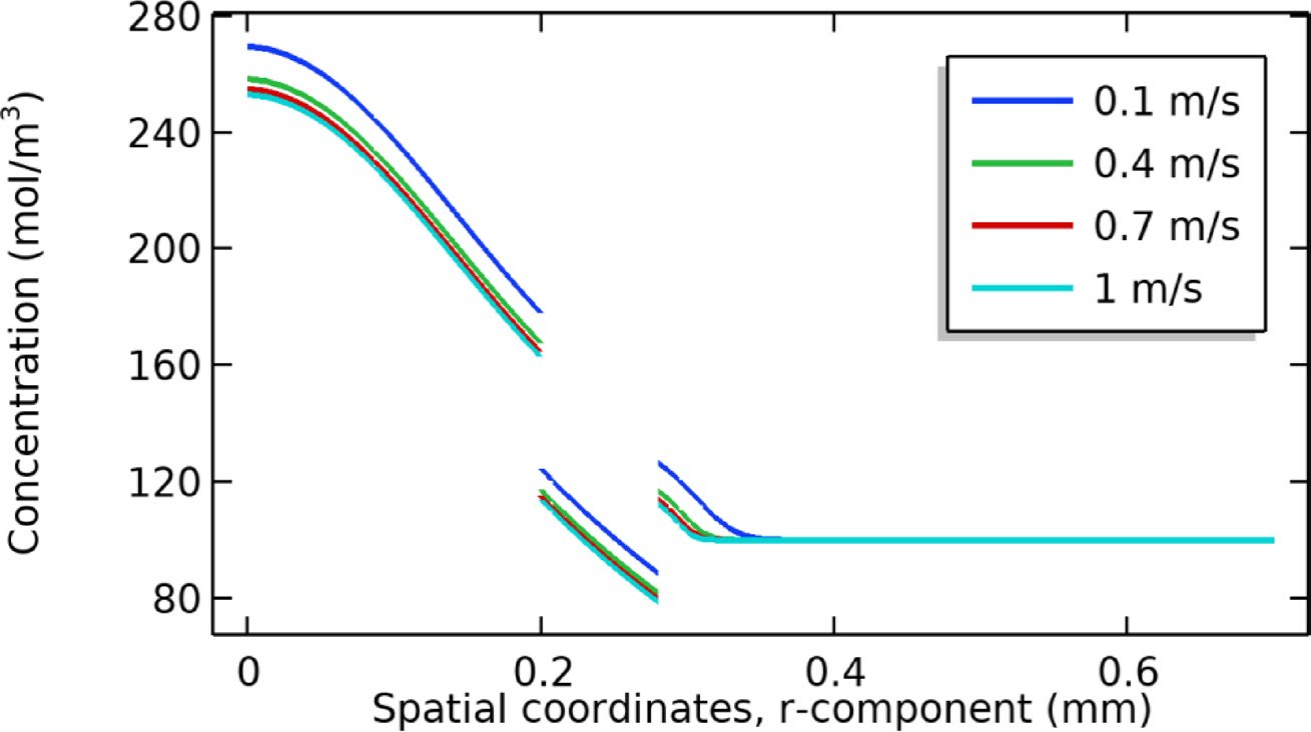
Shows the effect of permeate velocity on the concentration profile.

The effect of the permeate is studied by changing its values from 0.1 to 1 m/s. The results show that by increasing the velocity, the fluid passes quickly through the computational domain, and the concentration values decrease as speed increases for the dialysate and membrane parts. These results are presented in Fig. 12. Speed has no major effect on the permeate part, and its concentration profile remains the same or close to the same with minor changes. In the permeate part, it becomes a constant value close to 100 mol/m^3^. The overall trend is first decreasing in nature and then becomes constant. This is due to the reason that the pressure is driven when the solute passes the membrane surface boundary layer, and a polarization process occurs^34^. This results in dominance resistance at the feed side, as compared to the permeate side, which leads to a constant concentration profile. Also, in the permeate part, the fluid flow is diluted relatively, and fast convective homogenous mixing occurs. The equilibrium conditions are achieved quickly, and then even if there is a change in feeding velocity, there is a minor effect on the concentration profile, and hence it becomes constant after a certain time^35^^36^.

### Design of experiment (DOE) by Taguchi method

The design of experiments for testing the effect of the input variable on the concentration profile, using the Taguchi method for optimization with an array of L_16_ (4×4), as shown in Table 3, for fluid passes through it.

**Table 3:** Design of experiments for input parameters.

Exp #	d_c_ (Dialysate concentration)	K (Partition coefficient)	d_v_ (Dialysate velocity)	P_V_ (Permeate velocity)
1	0.1	0.1	0.1	0.1
2	0.1	0.4	0.4	0.4
3	0.1	0.7	0.7	0.7
4	0.1	1	1	1
5	0.4	0.1	0.4	0.7
6	0.4	0.4	0.1	1
7	0.4	0.7	0.7	0.4
8	0.4	1	0.7	0.4
9	0.7	0.1	0.7	1
10	0.7	0.4	1	0.7
11	0.7	0.7	0.1	0.4
12	0.7	1	0.4	0.1
13	1	0.1	1	0.4
14	1	0.4	0.7	0.1
15	1	0.7	0.4	1
16	1	1	0.1	0.7

### Analysis of variance (ANOVA) for concentration

In this statistical method, ANOVA analysis, which is a regression analysis carried out for the effect of input parameters on the concentration profile of the membrane. After doing a detailed analysis using Minitab software, the following results were obtained. The regression equation obtained from this analysis for concentration C_i_ in terms of all optimization parameters is

5 $${\text{C}}_{i}=813-47{\text{C}}_{\text{d}}+69\text{ K}-126{\text{D}}_{\text{v}}-337 {\text{P}}_{\text{v}}$$

Equation 5 confirms that the partition coefficients K have a significant impact on the results of the concentration profile, as their numerical coefficient is positive. In a negative numerical coefficient, the P_v_ is the second effective because of the high numerical coefficient, and so on_._

**Fig. 13:**
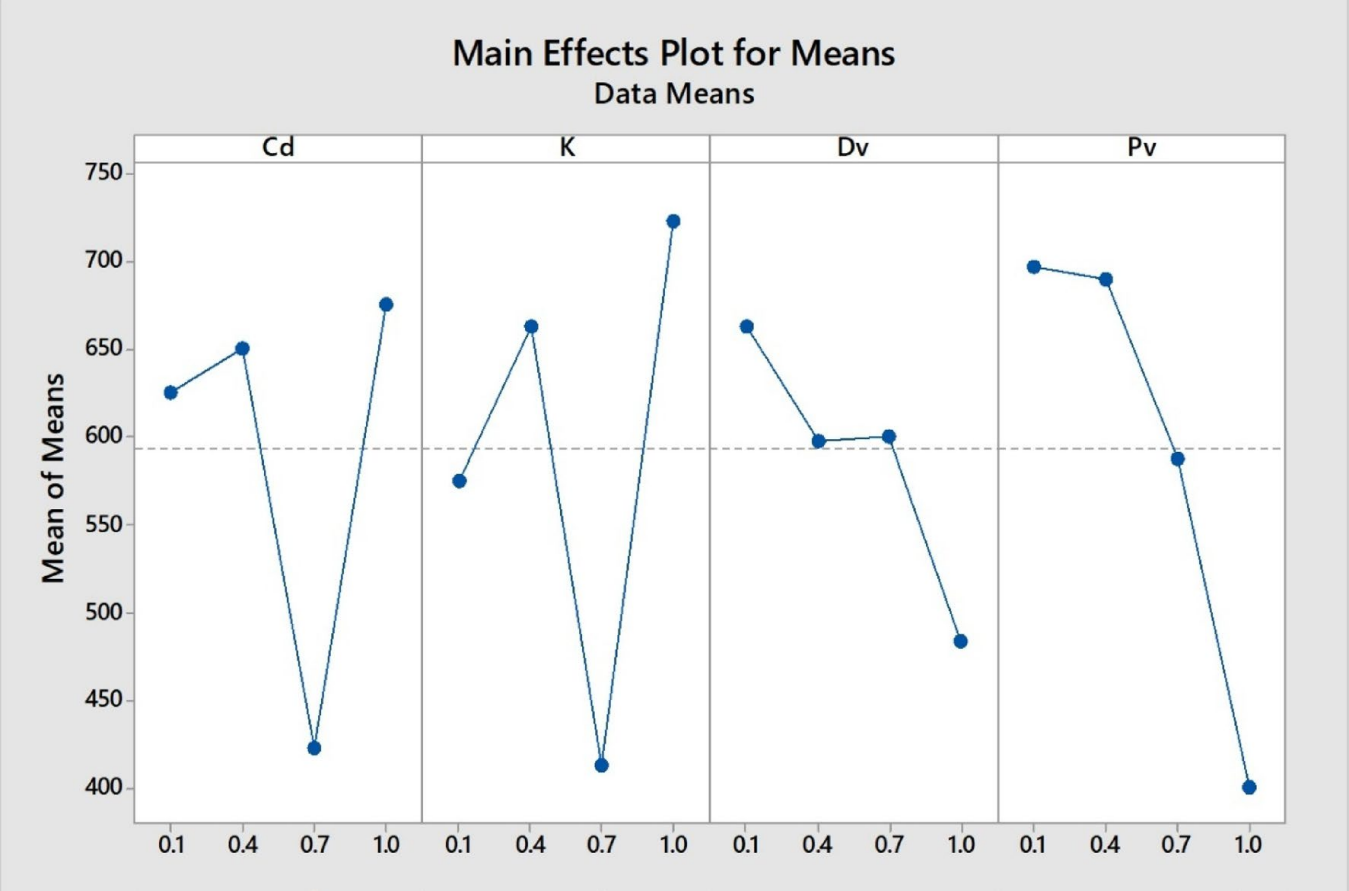
Shows the effect of permeate velocity on the concentration profile.

The main effect plot is represented in Fig. 13, showing the effects of different parameters on the concentration profile.

Table 4 shows the effect of different parameters on the performance or concentration profile. This can be written like this in mathematical form: K > P_v_ > C_d_ > D_v_. This means that the major effect is because of the partition coefficient, and the minor effect is due to permeate velocity.

**Table 4:** presents the experiment design for ranking each parameter’s effect on the concentration profile.

Level	C_d_	K	D_v_	P_v_
1	625	575	662.5	696.7
2	650	662.5	597.5	690
3	422.5	412.5	600	587.5
4	675	722.5	483.3	400
Delta	252.5	310	187.5	296.7
Rank	3	1	4	2

Table 5 represents the results of the ANOVA analysis for concentration. The columns of this table show the sources of variation versus degree of freedom (DF), Adj sum of squares (Adj SS), Adj mean of squares (Adj MS), statistics F-value, and the P-value. The last column of this table is for the sources’m percentage contribution. The P-value for C_d_ and K is given by 0.851 and 0.677, which is greater than 0.05, so these resources or parameters are not significant. But for D_v_ and P_v_, the P-values are respectively 0.480 and 0.076, showing significance. Also, the complete model summary is given by the same table, having S = 217.57, R-square 30.97%, R-square (adj) = 5.87%, and R-square (predicted) = 0.00%.

**Table 5:** ANOVA analysis for concentration.

Source	DF	Adj SS	Adj MS	F-value	P-Value
C_d_	1	1740	1740	0.04	0.851
K	1	8668	8668	0.18	0.677
D_v_	1	25,249	25,249	0.53	0.480
P_v_	1	180,952	180,952	3.82	0.076
Error	11	520,720	47,338		
Total	15	754,344			
Model Summary	S = 217.57	R-sq = 30.97%	R-sq (Adj) = 5.87%	R-sq (Pred) = 0.00%	

The design of the experiments table using the Taguchi method for ANOVA analysis helps in theoretically supporting and verifying the numerical results statistically. This analysis also helps in finalizing the parametric set of lists about which results are better. These analyses are very important for design engineers and give predictive results before fabrication and testing. The designer can set the dimensions of the geometry as per the desired results based on this analysis. It is best practice to do simulation analysis before experimental work to get predictive results. The optimization study can save much time and effort, as well as material and experimental costs for doing experiments for all cases. Once results are optimized, experimental fabrication and testing are conducted to get the desired level of results. Since this work is only a simulation for the best optimized design, future experimental studies will be conducted.

## Conclusion, limitations, and future work

This work focuses on membrane numerical modeling and simulation. The optimization of fluid flow profile is studied through the membrane by COMSOL 6.3 and the design of experiment Taguchi method with different input parameters. The results are validated against published papers, showing good agreement in results for validation of the model. Initial results are generated by setting initial values of the parameters in COMSOL 6.3. Then a range of values is defined, and their individual effects are studied on the concentration profile. Results show that the K parameter has a major impact on concentration, and P_v_ has a minor impact. For further verification, the design of experiments for an array of L_16_ (4×4) using Minitab software and its results again confirm the order K > P_v_ > C_d_ > D_v_ for the combined effect. The rank and main effect plots summarize the importance of parameter value variation. So, the major effect is due to the partition coefficient K, and the minor effect is that of permeate velocity as per analysis in Table 3. The current limitation of this work is that it cannot be fabricated with the current experimental setup, as the dimensions of the geometry are small and accuracy is very important. This work is limited to numerical study and is helpful for design improvement and optimization. In the future, the experimental setup will be established, and it will then be possible to fabricate and practically test it. This study helps in the prediction of the results, trends, and performance in terms of fluid flow concentration profile that can be affected by small changes in input parametric values. So, the study will be helpful for new researchers to have a better understanding of these effects while selecting the dimensions and input parameters list. The benefit is to save the cost of testing and fabrication by getting predictive simulation results, as it needs only computational power and skill. Another limitation is that parametric analysis for input parameters is performed, but full-scale optimization with additional advanced-level algorithms like grey analysis will be targeted in our future work.

## Supplementary Information

Below is the link to the electronic supplementary material.

Supplementary Material 1

## Data Availability

Major data generated or analyzed during this study are included in this article. However, for readers, COMSOL 6.3 and Minitab steps are included in the supplementary file.
