# Supplementary material for: Numerical parametric optimization of fluid flow profiles in membranes using the Taguchi method
Source: Sci Rep. 2025 Oct 14;15:35902. doi: 10.1038/s41598-025-19792-z (PMC12521374; doi:10.1038/s41598-025-19792-z)
Supplement: Supplementary file 1 — Supplementary Material 1 [file 41598_2025_19792_MOESM1_ESM.docx]

**Numerical Parametric Optimization of Fluid Flow Profiles in Membranes Using the Taguchi Method**

Muhammad Arslan^1^, Haizhen Xian^1^, Imran Shah^2^, Shahid Aziz^3^, Dong-Won Jung^4, *^

^1^School of Energy Power and Mechanical Engineering, North China Electric Power University

^2^Department of Mechatronics Engineering, Air University, Islamabad 44000, Pakistan

^3^Department of Mechanical Engineering, Jeju National University, 102 Jejudaehak-ro, Jeju-si 63243, Republic of Korea

^4^Faculty of Applied Energy System, Major of Mechanical Engineering, Jeju National University, 102 Jejudaehak-ro, Jeju-Si 63243, Republic of Korea

*Correspondence: authors: Dong-Won Jung ([jdwcheju@jejunu.ac.kr](mailto:jdwcheju@jejunu.ac.kr))

**COMSOL steps:**

1. **Geometry:** Open COMSOL, select the 2D axis symmetry
2. **Physics:** Add laminar flow and diluted concentration physics
3. **Steady study:** Select steady state conditions for this study
4. **Boundary conditions:** Add Dirichlet boundary conditions for velocity and concentration on the pressure on the outlet.
5. **Meshing:** Do meshing start with a fine mesh for better accuracy in results and try at least 5 different cases to select the optimal mesh. An optimal mesh is a mesh with enough accuracy and less computational cost.
6. **Running computation:** run the computation and wait for it to solve the problem with convergence. In case of divergence, the computation will give an error.
7. **Post processing:** extract line and surface plots directly or extract data from COMSOL and do it in other software like Origin Pro, Tec plots, etc.

**Minitab and ANOVA analysis:**

1. **Design of experiments:** based on the array selected, depending on the number of cases, factor levels, for example, in the current study, it L_16_ (4^4) indicates 16 experiments with 4 factors and 4 levels.
2. **Taguchi Analysis:** from the top menu, select the Taguchi analysis. It will prompt for the selection of inputs and responses, and then run for results
3. **Results:** Main effect plots, regression equation, and all data will be generated and can be summarized for ranking and delta calculation.
